# Depressive Symptom Profiles Predict Specific Neurodegenerative Disease Syndromes in Early Stages

**DOI:** 10.3389/fneur.2020.00446

**Published:** 2020-05-29

**Authors:** Suzanne M. Shdo, Kamalini G. Ranasinghe, Virginia E. Sturm, Katherine L. Possin, Brianne M. Bettcher, Melanie L. Stephens, Jessica M. Foley, Shou-Chin Christine You, Howard J. Rosen, Bruce L. Miller, Joel H. Kramer, Katherine P. Rankin

**Affiliations:** ^1^Memory and Aging Center, University of California, San Francisco, San Francisco, CA, United States; ^2^Department of Psychology, University of California, Berkeley, Berkeley, CA, United States; ^3^Department of Neurology, University of Colorado School of Medicine, Denver, CO, United States; ^4^Semel Institute for Neuroscience and Human Behavior, University of California, Los Angeles, Los Angeles, CA, United States

**Keywords:** depression, neurodegenerative disease, dysphora, hopelessness, worry, Alzheimer's, progressive supranuclear palsy, frontotemporal dementia

## Abstract

**Background:** During early stages, patients with neurodegenerative diseases (NDG) often present with depressive symptoms. However, because depression is a heterogeneous disorder, more precise delineation of the specific depressive symptom profiles that arise early in distinct NDG syndromes is necessary to enhance patient diagnosis and care.

**Methods and Findings:** Five-hundred and sixty four participants self-reported their depressive symptoms using the Geriatric Depression Scale (GDS), including 111 healthy older control subjects (NC) and 453 patients diagnosed with one of six NDGs who were at the mild stage of disease (CDR® Dementia Staging Instrument ≤ 1) [186 Alzheimer's disease (AD), 76 behavioral variant frontotemporal dementia (bvFTD), 52 semantic variant primary progressive aphasia (svPPA), 46 non-fluent variant PPA (nfvPPA), 49 progressive supranuclear palsy syndrome (PSPS), 44 corticobasal syndrome (CBS)]. The GDS was divided into subscales based on a previously published factor analysis, representing five symptoms (dysphoria, hopelessness, withdrawal, worry, and cognitive concerns). Mixed models were created to examine differences in depression subscale by group, and logistic regression analyses were performed to determine if patterns of depressive symptoms could predict a patient's NDG syndrome. PSPS patients presented with a hopeless, dysphoric, and withdrawn pattern, while patients with CBS presented with a similar but less severe pattern. Worry was a key symptom in the profile of patients with svPPA, while ADs only had abnormally elevated cognitive concerns. Depressive profile accurately predicted NDG diagnosis at a rate of between 70 and 84% accuracy.

**Conclusions:** These results suggest that attention to specific depressive symptom profile can improve diagnostic sensitivity and can be used to provide more individualized patient care.

## Introduction

Early stage neurodegenerative diseases (NDG) are often mistaken for primary psychiatric disorders, including major depressive and bipolar disorders (1). Furthermore, depressive symptoms are often comorbid with NDG (2). For example, one population study found the prevalence of depression in patients with corticobasal syndrome (CBS) to be 70% (3). Estimates of depressive comorbidity in patients with Alzheimer's disease (AD) syndrome are much more variable, with studies reporting prevalence rates ranging from 13 to 51% (4–6). Moreover, some NDG symptoms such as apathy and psychomotor slowing overlap phenomenologically with depression, which can confound estimates of comorbidity and make it difficult to parse apart syndromes.

Depression is itself a heterogeneous disorder. The DSM-V characterizes major depressive disorder as a mood disorder comprising affective, cognitive, and physical symptoms (7). Some symptoms of depression have more severe implications than others; for instance, studies in patients with major depressive disorder show that self-reported hopelessness is associated with increased suicidal ideation and risk of suicide attempts (8). Despite the heterogeneous nature of depression, and the differing clinical importance of distinct depressive symptoms, most studies of patients with NDG have measured depression as a unified entity, failing to characterize symptom profiles in a more nuanced way that may provide critical information about treatment and care to physicians and caregivers. Additionally, because early in disease progression patients with NDG have distinct, focal patterns of neural disruption, they provide a unique model for studying the brain-behavior relationships underpinning specific depressive symptoms. The aim of this study was to examine patterns of self-reported depressive symptomatology in a large cohort of early NDG patients with distinct clinically diagnosed neurodegenerative syndromes.

## Methods

### Participants

The 564 study participants included 111 healthy older controls (NC) and 453 patients. One-hundred and eighty-six patients met NINDS-ADRDA criteria for AD syndrome (9), 76 met FTDC criteria for behavioral variant frontotemporal dementia (bvFTD) (10), 52 met international criteria for semantic variant primary progressive aphasia (svPPA) (11) and 46 met criteria for non-fluent variant PPA (nfvPPA), 49 met criteria for progressive supranuclear palsy syndrome (PSPS) (12), and 44 met criteria for CBS (13). The study was approved by the Committee on Human Research at University of California, San Francisco. Patients were recruited through clinic referrals, all participants signed informed research consent, then their syndromic diagnosis was determined by a multidisciplinary consensus of a team based on neurological exam, history, neuropsychology, and structural MRI. Because we were interested in depressive symptom profiles early in the disease progression, patients were included only if they were in very early stages (CDR® Dementia Staging Instrument ≤1, “very mild” or “mild” level of functional impairment) All questionnaires were administered by trained neuropsychologists who confirmed the patient's ability to provide a valid self-report, including adequate language comprehension, cooperation, motivation, and insight.

Control participants were recruited through local advertisements, and were required to have an unremarkable neurological exam and structural MRI, and no functional or cognitive deficits on the basis of history and neuropsychological evaluation.

### Measures

The CDR® is a semi-structured interview, which measures functional impairment across six domains, including memory, orientation, judgment and problem solving, community affairs, home and hobbies, and personal care. Function scores for each question in each domain are based on a five point Likert scale with scores ranging from 0 to 4 (0 = none, 0.5 = very mild, 1 = mild, 2 = moderate, and 3 = severe) (14). Scores in each of these categories are combined to a composite score from 0 to 3. To ensure we were looking at early symptoms, only participants with a CDR® of 1 or lower (“very mild” or “mild”) were included in the study.

The Geriatric Depression Scale (GDS) is a 30-item self-report questionnaire, with categorical, “yes/no” response types. It is psychometrically validated to measure depression in older adults and patients with cognitive impairments (15). The GDS total score ranges from 0 to 30 with scores between 0 and 9 falling is scored as mild to moderate depression We subdivided the GDS into five distinct depressive symptom subscales, previously derived and validated by Adams et al. (16) via factor analysis. Subscales included: dysphoria (e.g., “Do you often feel downhearted and blue?”), hopelessness (“Do you feel that your situation is hopeless?”), withdrawal (“Do you prefer to avoid social gatherings?”), worry (“Do you frequently worry about the future?”), and cognitive complaints (“Is it easy for you to make decisions?”). Participants completed the questionnaire with the guidance of a neuropsychologist, who also provided confirmation patients' comprehension and valid participation in the procedure.

### Statistical Analysis

Potential between-group demographic confounds and disease severity [CDR® Sum of Boxes score (CDR®-SOB)] were evaluated using SAS proc GLM (version 9.4; SAS Institute Inc.), with a Dunnett-Hsu *post-hoc* test group comparisons to NCs.

A mixed design ANOVA was conducted to identify significant differences in patterns of depressive subscale scores among the six NDG groups, accounting for shared variance and multicollinearity. To identify subscales in which any NDG group was statistically different from controls or from any other NDG group, we conducted a mixed model analysis using raw subscale scores from all patient groups and controls, adjusting for age and sex, with subject identity included as the repeated factor, followed by an examination of the pair-wise comparisons of the least squared mean score (i.e., the mean adjusted for covariates) of each subscale across all groups. The significance threshold accounting for family-wise error was *p* < 0.00908 using the Benjamini–Yeketuli method (17).

To determine whether any NDG group's mean scores fell in a quantitatively clinically abnormal range, each NDG patient's scores were standardized to *z*-scores using the means and standard deviations from the NC cohort. A *z*-score cutoff of 1.35 (i.e., >91st percentile) was set as the threshold above which a group's mean score was considered abnormally clinically elevated.

Lastly, to determine if patient GDS subscale patterns were divergent enough to predict membership in specific NDG groups, we conducted six logistic regression analyses comparing each NDG group to a group comprised of all other NDG patients, controlling for age and gender. Standardized scores were used in these models to account for any between-subscale variance seen in controls. Odds ratios and 95% confidence intervals were calculated to determine which GDS subscales had the greatest influence in discriminating each group of NDG patients from all other patients, deriving classification accuracy, and percent concordance via leave-one-out cross-validation.

## Results

### Behavioral Results

Demographic characteristics of the participants are presented in Table 1. All NDG groups had elevated measures of disease severity (CDR® scale score, CDR® SOB score, and Mini Mental Status Examination score (*p* < 0.001 vs. NCs), but mean CDR® for all groups was <1 with very small standard deviations, indicating that patients in the groups all had very early/mild dementia and there were no differences in disease severity that could explain other results (Table 1). The total GDS score for all NDG groups was significantly higher than NCs (AD, bvFTD, svPPA, CBS, PSPS *p* < 0.001; nfvPPA *p* < 0.01). Pairwise comparisons revealed significant diagnostic group differences in the GDS total scores. Individuals with PSPS had significantly higher GDS total scores than individuals with AD, bvFTD, and nfvPPA (*p* < 0.001). Patients with CBS and svPPA had significantly higher scores than individuals with AD and nfvPPA (*p* < 0.001). Of the 453 cases, 153 met the criteria for depression based on the GDS total score alone.

**Table 1:** Participant demographics.

	**PSPS**	**CBS**	**bvFTD**	**svPPA**	**nvfPPA**	**AD**	**NC**		
	**(*n* = 49)**	**(*n* = 44)**	**(*n* = 76)**	**(*n* = 52)**	**(*n* = 46)**	**(*n* = 186)**	**(*n* = 111)**	**Test statistics**	***p*-value**
Age	68.6 (6.5)	67.8 (8.1)	60.1^**^ (7.8)	63.7^**^ (7.5)	68.0 (7.3)	64.7^**^ (9.7)	70.7 (9.0)	*F* (6, 563) = 14.21	*p* < 0.0001
Sex (M/F)	26/23	19/25	51/25	27/25	16/30	91/95	57/54	χ2 (6, 563) = 14.23	*p* = 0.0272
Education	16.2^*^ (3.8)	16.0^*^ (3.0)	16.1^**^ (3.2)	17.0 (2.7)	16.3 (3.5)	16.3^**^ (2.8)	17.6 (2.2)	*F* (6, 545) = 3.35	*p* = 0.003
MMSE	26.2^**^ (3.1)	24.5^**^ (4.2)	25.8^**^ (3.8)	24.6^**^ (4.5)	25.1^**^ (4.6)	22.4^**^ (5.0)	29.1 (1.2)	*F* (6, 563) = 32.53	*p* < 0.0001
CDR® (Total)	0.7^**^ (0.3)	0.6^**^ (0.3)	0.8^**^ (0.3)	0.6^**^ (0.2)	0.4^**^ (0.3)	0.7^**^ (0.3)	0.0 (0.0)	*F* (6, 563) = 118.53	*p* < 0.0001
CDR® (SOB)	4.4^**^ (2.3)	3.0^**^ (2.0)	5.3^**^ (2.0)	3.8^**^ (2.2)	1.7^**^ (1.6)	3.9^**^ (2.1)	0.0 (0.1)	*F* (6, 563) = 83.3	*p* < 0.0001
GDS total	12.7^**^ (7.2)	10.0^**^ (6.1)	8.5^**^ (6.8)	9.7^**^ (6.5)	6.4^*^ (4.9)	6.9^**^ (4.8)	3.5 (4.1)	*F* (6, 563) = 8.57	*p* < 0.0001

*AD, Alzheimer's disease; bvFTD, behavioral variant frontotemporal dementia; CBS, corticobasal syndrome; CDR® (SOB), CDR® Dementia Staging Instrument (sum of boxes) (out of 16 points, and higher scores depict greater impairment); CDR® total, CDR® Dementia Staging Instrument total score (0–3 range, with 0 indicating no impairment); GDS, Geriatric Depression Scale (out of 30 points, and higher scores indicate greater depression); MMSE, Mini-Mental State Examination (out of 30 points, and higher scores depict better performance); NC, normal control; nfvPPA, non-fluent variant primary progressive aphasia; PSPS, progressive supranuclear palsy syndrome; svPPA, semantic variant primary progressive aphasia. For age, education, MMSE, CDR®, CDR® (SOB), and GDS, the numbers indicate mean (standard deviation). For sex the numbers indicate frequency of male/female sex identification*.

* *raw adjusted LS means differed from controls at p < 0.01*.

** *raw adjusted LS means differed from controls at p < 0.001*.

### Patterns of Depressive Symptoms by Diagnostic Group

Mixed model analysis controlling for age and gender revealed an interaction between diagnosis and GDS subscale, indicating statistically significant differences in pattern of depressive symptoms across NDG patient groups (*F* = 10.41, *p* < 0.001).

#### Progressive Supranuclear Palsy Syndrome (PSPS)

**Figure 1:**
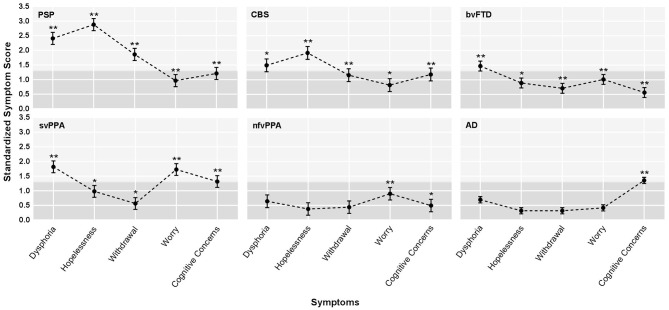
Profiles of average depression symptoms for the six dementia groups. AD, Alzheimer's disease syndrome; bvFTD, behavioral variant frontotemporal dementia; CBS, corticobasal syndrome; nfvPPA, non-fluent variant primary progressive aphasia; PSPS, progressive supranuclear palsy syndrome; svPPA, semantic variant primary progressive aphasia. Standardized depressive symptom score by diagnostic group. The graphs show the adjusted least square means (LS means) corrected for age and sex, and the standard errors, derived from the mixed-model analysis, based on *z*-score estimates calculated using older normal controls. The light gray shading indicates the range in which *z*-scores were considered clinically abnormal (*z* > 1.35). The stars indicate statistically significant differences between each patient subgroup and healthy older controls, derived from the mixed model analysis of raw scores. *adjusted LS means of raw scores differed from controls after family wise error correction at a threshold of *p* < 0.00908. **adjusted LS means raw scores differed from controls at *p* < 0.001.

Patients with PSPS reported significantly higher scores across all depressive symptom subscales when compared to controls (FWE threshold *p* < 0.00908 according to B–Y correction, see section Methods), and these differences reached the level of clinical significance (i.e., group mean score > 91st percentile compared to controls) for hopelessness (*z* = 2.87), dysphoria (*z* = 2.43), and withdrawal (*z* = 1.86; Figure 1). Also, hopelessness and withdrawal scores were significantly higher in the PSPS group than all other NDG patient groups, and patients with PSPS had higher dysphoria scores than patients with nfvPPA or AD. The PSPS group also had significantly higher cognitive concerns scores than bvFTD and nfvPPA patients (Table 2). In a binary logistic regression analysis using GDS subscales, age, and sex to contrast patients with PSPS with all other patients, 85% of patients were correctly classified after cross-validation (Wilks lambda = 0.82, *p* < 0.001), where higher hopelessness [MLE = 0.86, *p* < 0.001, 95% CI: (1.6–3.5)], higher withdrawal [MLE = 0.74, *p* < 0.005, 95% CI: (1.4–3.2)], and lower worry [MLE = −4.3, *p* < 0.05, 95% CI: (0.4–1.0)] significantly predicted PSPS group membership (Table 3).

**Table 2:** Mixed model, between-group analysis, including diagnostic groups that had statistically different scores on a symptom when compared to each other diagnostic group.

	**NC**	**PSPS**	**CBS**	**bvFTD**	**nfvPPA**	**svPPA**
	**(*n* = 111)**	**(*n* = 49)**	**(*n* = 44)**	**(*n* = 76)**	**(*n* = 52)**	**(*n* = 186)**
**Dysphoria**						
PSPS	5.69^**^					
CBS	3.27^*^	–				
bvFTD	3.56^**^	–	–			
nfvPPA	–	−3.67^*^	–	–		
svPPA	4.15^**^	–	–	–	–	
AD	–	−4.60^**^	–	–	–	−3.00^*^
**Hopelessness**						
PSPS	9.64^**^					
CBS	6.06^**^	−2.75^*^				
bvFTD	3.02^*^	−6.39^**^	−3.21^*^			
nfvPPA	–	−7.21^**^	−4.31^**^	–		
svPPA	3.06^*^	−5.67^**^	−2.73^*^	–	–	
AD	–	−9.44^**^	−5.65^**^	–	–	–
**Withdrawal**						
PSPS	9.06^**^					
CBS	5.33^**^	−2.90^*^				
bvFTD	3.72^**^	−5.27^*^	–			
nfvPPA	–	−5.94^**^	−2.93^*^	–		
svPPA	2.62^*^	−5.55^**^	–	–	–	
AD	–	−8.23^**^	−4.3^**^	–	–	–
**Worry**						
PSPS	3.69^**^					
CBS	2.98^*^	–				
bvFTD	4.32^**^	–	–			
nfvPPA	3.35^**^	–	–	–		
svPPA	6.9^**^	2.7^*^	3.11^*^	2.81^*^	2.87^*^	
AD	–	–	–	−3.04^*^	–	−5.90^**^
**Cognitive concern**						
PSPS	7.18^**^					
CBS	6.79^**^	–				
bvFTD	3.71^**^	−3.55^**^	−3.33^**^			
nfvPPA	2.73^*^	−3.66^**^	−3.47^**^	–		
svPPA	8.08^**^	–	–	4.44^**^	4.40^**^	
AD	11.57^**^	–	–	6.08^**^	5.63^**^	–

*AD, Alzheimer's disease; bvFTD, behavioral variant frontotemporal dementia; CBS, corticobasal syndrome; nfvPPA, non-fluent variant primary progressive aphasia; PSPS, progressive supranuclear palsy syndrome; svPPA, semantic variant primary progressive aphasia. Numbers depict the between group t-values derived from the mixed model analysis; Positive t-values indicate that the score of patient group in the left column is greater than the corresponding patient group in the top row. Negative t-values indicate that the score of patient group in the left column is lower than the patient group on the top row*.

* *raw adjusted LS means differed from controls at p < 0.01*.

** *raw adjusted LS means differed from controls at p < 0.001; –, not significant*.

**Table 3:** Logistic regression analysis, maximum likelihood estimates, 95% confidence intervals, and *p*-values for each diagnostic group compared to all other neurodegenerative disease diagnoses.

		**PSPS**	**CBS**	**bvFTD**	**svPPA**	**nfvPPA**	**AD**
		**(*n* = 49)**	**(*n* = 44)**	**(*n* = 76)**	**(*n* = 52)**	**(*n* = 46)**	**(*n* = 186)**
% Concordant		84.7%	70.1%	79.2%	69.7%	75.7%	76.0%
Dysphoria	MLE	−0.13	−0.31	0.23	0.33	−0.47	0.00
	95% CI	0.6–1.3	0.5–1.1	0.9–1.9	0.9–2.1	0.4–1.1	0.7–1.4
Hopelessness	MLE	0.86^**^	0.56^**^	−0.06	−0.29	−0.38	**−0.52**
	95% CI	**1.6–3.5**	**1.2–2.5**	0.7–1.4	0.5–1.1	0.4–1.1	**0.4–0.8**
Social withdrawal	MLE	0.75^**^	0.25	0.21	**−0.46**	0.13	**−0.46**
	95% CI	**1.4–3.2**	0.9–1.9	0.9–1.7	**0.4–1.0**	0.8–1.7	**0.5–0.8**
Worry	MLE	−0.43^*^	−0.14	0.13	0.64^*^	0.63^**^	−0.41^**^
	95% CI	**0.4–1.0**	0.6–1.3	0.9–1.9	**1.4–2.6**	**1.3–3.0**	**0.5–0.9**
Cognitive concerns	MLE	−0.32	−0.06	−0.88^**^	0.18	−0.65^**^	0.92^**^
	95% CI	0.5–1.1	0.6–1.4	**0.9–0.6**	0.8–1.7	**0.4–0.8**	**1.9–3.7**

*AD, Alzheimer's disease; bvFTD, behavioral variant frontotemporal dementia; CBS, corticobasal syndrome; nfvPPA, non-fluent variant primary progressive aphasia; PSPS, progressive supranuclear palsy syndrome; svPPA, semantic variant primary progressive aphasia. MLE, maximum likelihood estimate; 95% CI, confidence interval. Percentages represent the fraction of patients correctly classified by logistic regressions which compared each diagnostic group with all of the other neurodegenerative disease groups using the standardized symptom scores (z-scores). The values represent the maximum likelihood estimates, 95% confidence intervals (CIs) associated with odds ratios, and p-values. Along each column, the bold text indicates the symptom categories with the greatest influence on the model for the respective neurodegenerative disease group as compared to all other neurodegenerative diseases*.

* *p < 0.05*,

** *p < 0.005*.

*We have highlighted the fields to be emphasized, ideally bolded*.

#### Semantic Variant Primary Progressive Aphasia (svPPA)

Patients with svPPA had significantly higher scores on all GDS subscales than NCs, with the average dysphoria (*z* = 1.82) and worry (*z* = 1.73) scores falling in the clinically abnormal range (Figure 1). The svPPAs were the only NDG group with abnormally high worry, significantly higher than all other NDG groups (Table 2). Patients with svPPA also had significantly higher dysphoria scores than ADs, and cognitive concerns scores than patients with bvFTD and nfvPPA. A model including higher worry [MLE = 0.64, 95% CI: (1.4–2.6); *p* < 0.001] and lower withdrawal [MLE = −0.46, 95% CI: (0.4–1.0); *p* = 0.03] scores distinguished svPPAs from other patients at 70% accuracy (Wilks lambda = 0.93, *p* < 0.0001 (Table 3).

#### Corticobasal Syndrome (CBS)

Compared to the NC's, patients with CBS had significantly higher scores on all GDS subscales, although only hopelessness (*z* = 1.92) and dysphoria (*z* = 1.49) scores fell in the clinically abnormal range (Figure 1). When compared to all other NDG patient groups, patients with CBS had significantly higher hopelessness scores than all other patients except for the PSPS group (bvFTD, svPPA, nfvPPA, and AD; Table 2). High hopelessness significantly predicted CBS group membership at 70% accuracy [MLE = 0.56, 95% CI: (0.6–1.3); *p* = 0.004; Table 3].

Patients with bvFTD had significantly higher scores than NCs on all GDS subscales, though only dysphoria scores were abnormally clinically elevated (*z* = 1.48; Figure 1) and reported significantly higher worry than ADs (Table 2). Lower reported cognitive concerns predicted bvFTD group membership [MLE = −0.88, 95% CI: (−0.88 to 1.35); *p* < 0.0001; Table 3], in a model classifying 79% of patients.

#### Alzheimer's Disease (AD)

In comparison with NC's patients with AD syndrome only had significantly higher scores on the cognitive concern subscale. Additionally, patients with AD were the only NDG patient group in which cognitive concerns were abnormally elevated (*z* = 1.35; Figure 1, Table 2). Seventy-six percent of patients with AD were correctly discriminated from other patients by their high cognitive concerns [MLE = 0.92, 95% CI: (1.9–3.7); *p* < 0.0001], and lower worry [MLE = −0.41, 95% CI: (0.5–0.9); *p* < 0.01], withdrawal [MLE = −0.46, 95% CI: (−0.46 to 0.8); *p* < 0.001], and hopelessness [MLE = −0.52, 95% CI: (0.4–0.8); *p* < 0.001; Table 3].

### Non-fluent Variant Primary Progressive Aphasia (nfvPPA)

Patients with nfvPPA were significantly higher than NCs on worry and cognitive concerns scores, but no symptom was abnormally elevated (Figure 1), and nfvPPAs had no significant differences from other NDG groups (Table 2). Classification modeling correctly classified 76% of patients into nfvPPA vs. other NDG groups, where higher worry [MLE = 0.63, 95% CI: (1.3–3.0); *p* < 0.01] and lower cognitive concerns [MLE = 0.65, 95% CI: (1.3–3.0); *p* < 0.01) significantly predicted syndrome (Table 3).

## Discussion

We found distinct patterns of self-reported depressive symptoms in the early stages of six NDG syndromes. We then used those profiles to predict syndrome class without additional clinical features. Our findings suggest that more precise characterization of depressive symptom profiles, early in the disease process, may contribute a new domain of clinical information that improves differential diagnosis among NDG syndromes. While our study was not designed to definitively identify the etiology of the depressive symptoms reported by the patients, these results point toward a biological rather than environmental cause. All of these individuals were similarly diagnosed with a presumably fatal neurodegenerative disorder, and were coping with the impact on their lives of new functional changes; however, the symptom patterns across the syndrome groups varied significantly, suggesting that the endorsement or denial of symptoms was at least partly associated with specific disease processes, rather than reflecting a general reaction to external stressors. Thus, these results suggest that further investigation of the disease-specific biologic mechanisms underlying these psychiatric features is warranted.

### Prominent Depressive Symptom Profiles by Syndrome

Our patients with PSPS and CBS had elevated depression early in their disease, but we extend the existing literature (2, 18) by showing that hopelessness is a primary feature of their depressive symptom profiles. Patients with PSPS report levels of hopelessness ~3 standard deviations higher than the average healthy older adult (CBS was 2.5 SDs higher). In a review of behavioral symptoms of PSPS, Gerstenecker et al. (19) found a majority of relevant studies reported elevated depression in CBS, however all but one study reported that the predominant psychiatric symptom in PSP was apathy, and indicated that PSP patients were not depressed (20). Differences in reporting modality may explain this discrepancy; the one study finding patients with PSPS had elevated depression used a self-report measure, consistent with our approach, while all others relied on caregiver reports. Together, these results suggest that in PSPS patients, internalized symptoms of depression like hopelessness and sadness may not be reliably observed by external observers, including caregivers, and apathy and depression can look similar to an outside observer. Our use of a patient self-report approach ameliorated this problem, because if patients were apathetic they would be likely to not explicitly endorse depressive thoughts and feelings, thus in this context symptom endorsements suggest that the patients are actually experiencing depression rather than apathy. Magnitude of hopelessness may also help diagnostically classify PSPS and CBS patients at the earliest stages; in our sample, the level of hopelessness seen in PSPS was significantly higher than the already clinically elevated scores of patients with CBS. PSPS and CBS are both characterized by early degeneration of brain regions related to reward and emotion (e.g., basal ganglia, other subcortical structures) (21, 22) thus further study is needed to determine the role these structures play in the development of psychiatric symptoms.

Primary characteristics of bvFTD include apathy and withdrawal, but previous studies have suggested marked changes in mood are rare (23), and the mild elevations in depression found using caregiver reports like the Neuropsychiatric Inventory (NPI) are no higher than other NDG groups (24, 25). Our study confirmed that mild elevations in multiple depressive symptoms may be fairly common in early bvFTD self-reports. Dysphoria was also abnormally elevated, suggesting the presence and impact of this symptom should be routinely investigated in the clinical care of early bvFTD patients, though some reports suggest that initially higher depression scores in these patients may decrease with disease progression (26).

Another key finding was that pathological worry is a prominent, unique hallmark of svPPA, warranting consideration in both differential diagnosis and clinical care. Questions on the GDS worry subscale include, “I worry a lot about the past; I frequently worry about the future; I am afraid that something bad is going to happen to me.” Other studies report that patients with svPPA have increased mental rigidity, compulsions, distress, and anxiety when confronted by challenges (27, 28), thus behavioral interventions targeted to mitigate excessive worry should be considered in clinical care of patients with svPPA. Furthermore, svPPA patients are the only group in this study with predominantly left temporal lobe atrophy, which has implications for the mechanistic role of this region in excessive worry. Semantic loss and the attendant mental rigidity that are primary signs of anterior temporal disease (29) might reduce svPPA patients' ability to engage in healthy, flexible cognitive reappraisal of future events, a key emotion regulation strategy.

As a group, patients with early AD did not self-report clinically significant elevations in most depression symptoms, only reporting elevated concerns their cognition was worsening. This contrasts with other studies reporting higher prevalence of depression in AD, but there are several potential reasons for this discrepancy. First, only patients with mild AD were included in our study, while many other studies reporting higher levels of depression in AD include patients with a greater range of severity and more advanced disease progression (4, 5). There is some evidence that AD patients begin to progressively lose their ability to regulate negative emotions as early as the MCI stage, partly associated with right lateral temporal degeneration (30). Another source of difference might be that many studies measure depression in AD via informant reports (25, 31) which vary in reliability depending on the closeness and emotional sensitivity of the informant.

### Generalizability to Other Psychiatric Populations

These results also have more general implications for the functional anatomy of specific depressive symptoms in non-NDG populations. Measuring specific symptoms rather than depression as a whole is consistent with the National Institute of Mental Health (NIMH), Research Domain Criteria (RDoC) framework, which aims to better classify mental health disorders based on observable and neurobiological measures (32). Because the anatomic patterns of neurodegeneration in these syndromes are well-understood, particularly early in the disease process, psychiatric data from NDG patients may support generation of focal lesion models testing the specific role of neural circuits in particular depressive symptoms (33). For example, our finding that patients with PSP and CBS had increased risk of hopelessness and dysphoria supports the theory that subcortical regions, which are targeted early in both of these diseases, plays an important role in these symptoms, not only in NDG patients but more generally across psychiatric cohorts. Similarly, our finding that patients with svPPA, who have predominately left temporal atrophy, had markedly increased worry suggests that the left temporal lobe may play an important role in regulating processes that inhibit anxiety.

### Clinical Implications for Diagnosis and Management of NDG

These findings suggest that more precise evaluation of a patient's specific depressive phenotype may in fact aid in differential diagnosis, particularly in cases where the canonical cognitive and neurologic symptoms fail to clearly classify the patient. We found that apart from any other clinical symptoms, depressive profile alone can accurately predict diagnosis at a rate of between 70 and 84% accuracy with all but nfvPPA patients, whose depressive symptoms were clinically unremarkable. This suggests that administration of a depression measure that provides subscale scores, like the GDS, during standard NDG diagnostic evaluations could be a valuable adjunct to current assessment protocols. If these early psychiatric features are confirmed in other patient samples, it may also be appropriate to consider the addition of psychiatric features to current consensus diagnostic criteria.

Understanding differential patterns of depressive symptoms in individual NDG patients is also critical for optimal patient care. Behavioral management and pharmacology differs for, e.g., severe hopelessness and dysphoria than for a euthymic patient reporting excessive worry. Hopelessness is a severe symptom associated with increased suicidal ideation and suicidality in the general population (34). We did not directly measure suicidal ideation, and epidemiologic prevalence rates remain uncertain, but in one Japanese mixed dementia cohort, 10.1% of patients reported suicidal ideation (35). Several case studies have identified suicidal ideation in patients with PSPS (36, 37) and there are three published reports of completed suicide in this group (38, 39). Similarly, patients with svPPA reported clinically elevated levels of worry, emphasizing the value of thoroughly assessing and managing anxiety early on when developing individualized treatment plans. The high prevalence of psychiatric distress revealed by this study suggests that the field needs more treatment efficacy studies leading to the development of evidence-based, effective psychiatric interventions designed for patients with different NDG syndromes.

### Limitations and Future Directions

There are several limitations to this study that should be considered. First, the GDS is a self-report measure of depression, thus due to the inherent potential for self-report bias, future studies should confirm the existence of these early syndrome-specific depression patterns by harmonizing across observer-based and objective behavioral depression measures. Second, because the GDS is designed for an older population assumed to have multiple biological changes and medical comorbidities associated with aging (40) the GDS does not examine many key vegetative symptoms of depression (sleep, weight, appetite, and psychomotor), or other key cognitive-emotional symptoms such as rumination, which has been identified as a key mediator of depression (41). Examining these features may enhance understanding of early syndrome-specific depressive profiles. Third, because the focus of our paper was on only a subset of neurodegenerative syndromes, we were not able to examine all dementia syndromes, and comparison of depressive profiles in additional disorders such as Parkinson's disease dementia and vascular dementia will be valuable work in future studies. Similarly, while we included a healthy older adult control group, we did not have a depressed older adult cohort. A direct comparison with a depressed control group will be important for future studies to demonstrate differences between NDG symptom profiles and patterns of depressive symptoms in older adults with depression. It is also important to note that this normal control group, as well as the patients, were representative of older adults at our clinic, and thus it would be valuable for future studies to determine if these patterns are replicated in other samples. Another limitation is that because this is an observational, cross-sectional study, it is not possible to determine if these depressive symptom profiles are associated with neurologic changes or if the reaction to the diagnosis of a fatal illness. While our study was designed only to reveal depression profiles at the earliest stage of dementia, it will be important for future studies to observe patients throughout their disease course to determine how these profiles change. Examination of mechanisms associated with these unique symptom profiles will not only clarify the clinical presentation of patients with NDG, but may reveal key insights into the neural etiology of specific depressive symptoms in other cohorts.

## Data Availability Statement

The datasets generated for this study are available on request to the corresponding author.

## Ethics Statement

The studies involving human participants were reviewed and approved by UCSF Institutional Review Board. The patients/participants provided their written informed consent to participate in this study.

## Author Contributions

SS, KGR, VS, BM, and KPR contributed conception and design of the study. SS organized the data. SS and KPR performed the statistical analysis. SS wrote the first draft of the manuscript. KPR advised and significantly contributed to the writing of the manuscript. All authors contributed to manuscript revision, read, and approved the submitted version.

## Conflict of Interest

The authors declare that the research was conducted in the absence of any commercial or financial relationships that could be construed as a potential conflict of interest.
